# Current Salivary Glands Biopsy Techniques: A Comprehensive Review

**DOI:** 10.3390/healthcare10081537

**Published:** 2022-08-14

**Authors:** Matteo Pellegrini, Federica Pulicari, Paolo Zampetti, Andrea Scribante, Francesco Spadari

**Affiliations:** 1 Section of Dentistry, Department of Clinical, Surgical, Diagnostic and Pediatric Sciences, University of Pavia, 27100 Pavia, Italy; 2 Maxillo-Facial and Odontostomatology Unit, Department of Biomedical Surgical and Dental Sciences, Fondazione IRCCS Ca’ Granda Ospedale Maggiore Policlinico, University of Milan, 20122 Milan, Italy

**Keywords:** biopsy, fine-needle aspiration biopsy, frozen sections, imaging guided biopsy, minor salivary glands, salivary glands

## Abstract

Biopsy is a surgical procedure performed to collect a portion of tissue or organ for diagnostic studies. The aim of the present manuscript is to describe state-of-the-art major and minor salivary gland biopsy techniques and assess the indications and complications of other salivary gland biopsy techniques. A search was performed using the following MeSH terms: biopsy, fine-needle biopsies, image-guided biopsies, frozen sections, and salivary glands disease. A current overview of major and minor salivary glands biopsy techniques was provided. In the oncological field, a comparison was made between the most widely used biopsy method, ultrasound-guided fine-needle aspiration biopsy (US-FNAB), and an alternative method, ultrasound-guided core needle biopsy (US-guided CNB), highlighting the advantages and disadvantages of each. Finally, intra-operative frozen sections (IOFSs) were presented as an additional intraoperative diagnostic method. Minor salivary gland biopsy (MSGB) is the simplest diagnostic method used by clinicians in the diagnosis of inflammatory and autoimmune diseases. In neoplastic lesions, US-FNAB represents the most performed method; however, due to its low diagnostic accuracy for non-neoplastic specimens, US-guided CNB has been introduced as an alternative method.

## 1. Introduction

Biopsy is a surgical procedure performed to collect a portion of tissue or organ for diagnostic studies, which must be properly preserved and processed through marking systems and then histopathologically examined [1].

Salivary gland biopsy can be performed to diagnose diseases of salivary parenchyma, such as degenerative and inflammatory diseases, or amyloidosis and sarcoidosis [2]. This biopsy can be performed using simple and minimally invasive methods, such as minor salivary gland biopsy (MSGB); in particular, lower lip mucosa is considered to be the least invasive collection site for the collection of an adequate diagnostic sample [1].

Common histopathological findings, indicative of Sjogren’s syndrome, are represented by clusters of monocellular immune cells which are present in the exocrine glandular tissues and are responsible for atrophy [3]. The importance of conducting a histopathological examination of the minor salivary glands is underlined by the inclusion of a positive biopsy in the diagnostic criteria proposed by the American-European Consensus Group (AECG) [3].

MSGB is also effective to help diagnose amyloidosis, representing a much less invasive and faster method than kidney biopsy [4,5]. Furthermore, the MSGB method saves time on the diagnosis of the pathology, which can quickly lead to death in the case of diagnostic delay [6]. 

Another biopsy method used is the open technique, which can be variably complex and invasive, and involves various secreting tissues; they are considered surgical procedures, not free from potential local and systemic complications [7], such as paresthesias, intra- and postoperative bleeding, pain, local postoperative discomfort, swelling, ecchymosis, surgical wound infection, suture dehiscence, and, finally, keloid development [8]. A reduction of intra- and postoperative patient discomfort can be achieved by following anatomical and surgical criteria while ensuring an adequate specimen to perform microscopic diagnosis [9]. This type of biopsy can be useful for both incisional and excisional sampling, and it involves major salivary glands.

There are biopsy techniques mainly indicated in the oncology field, such as ultrasound-guided fine-needle aspiration biopsy (US-FNAB) and ultrasound-guided core needle biopsy (US-guided CNB), which could be useful in the preoperative diagnosis of benign and malignant neoplasms and pleomorphic adenomas [10].

However, due to the histological complexity of some neoplasms, it is difficult to diagnose the exact nature of salivary lesions. In these clinical conditions, intra-operative frozen sections (IOFSs) offer the best guarantee of optimal histologic results because of the possibility of evaluating the tissue before the end of the surgery, thus differentiating malignant from benign lesions and performing more aggressive surgery at once [11].

This comprehensive review aims to present the state-of-the-art major and minor salivary gland biopsy techniques, evaluating their indications and complications. Finally, the discussion is completed by presenting US-guided CNB, US-FNAB, and IOFSs.

## 2. Materials and Methods

### 2.1. Focused Questions

What are the feasible open salivary gland biopsy techniques? Are postoperative complications related? Are there other biopsy techniques for the salivary glands?

### 2.2. Eligibility Criteria

The following inclusion criteria guided the analysis of the studies:

Type of studies. Clinical trials, case-control studies, cross-sectional studies, cohort studies, narrative reviews, and systematic reviews.

Type of participants. Patients with SS, patients with immune-mediated or inflammatory diseases, and patients with benign or malignant neoplastic diseases. 

Type of interventions. Assessment of salivary gland biopsy techniques, with reference to minor salivary gland biopsy techniques, evaluated through case-control, cross-sectional, cohort, clinical, and review studies.

Outcome type. Indications, advantages, limitations, and complications of major and minor salivary gland biopsy techniques. Only studies that met all inclusion criteria were included. However, the following exclusion criteria were included: (I) abstract of articles published in non-English languages, (II) duplicate studies, (III) in vitro or animal clinical studies, (IV) not pertinent studies, and (V) absence of Ethics Committee approval.

### 2.3. Search Strategy

The PICO model (population, intervention, comparison, outcome) was used to perform this review, through a literature search of the PubMed (MEDLINE) and Scopus electronic databases. Abstracts of studies that evaluated the indications, advantages, limitations, and complications of salivary gland biopsy techniques were reviewed. 

### 2.4. Research

The medical subject heading (MeSH) terms are: biopsy, fine-needle biopsies, image-guided biopsies, frozen sections, and salivary glands disease; an electronic search was carried out with PubMed (MEDLINE) and Scopus databases. Articles published in the years from 1960 to 2022 were targeted. The duration of data extraction was between 10 and 12 weeks. The last search was performed on 4 July 2022. Two calibrated reviewers (M.P. and F.P.) performed the search. Disagreements and discrepancies were resolved by consensus and two other reviewers were consulted (P.Z. and F.S.). All the titles and abstracts were read thoroughly from the articles primarily searched, and nonrelevant studies were excluded. The relevant articles were enlisted and scrutinized for any similar studies which matched our inclusion criteria. For the extraction of pertinent results, we read full texts of the included studies and the findings were recorded.

### 2.5. Quality Assessment of Included Studies

A methodological quality risk of bias assessment was used to perform this review.

A quality analysis for in vivo studies was conducted through the National Heart, Lung and Blood Institute (NIH) Quality Assessment Tool for Before-After (Pre-Post) Studies with No Control Group, for Observational Cohort, Cross-Sectional Studies and Case-Control Studies, and for Systematic Reviews and Meta-Analyses [12], the Methodological Index for Non-Randomized Studies (MINORS) criteria [13], and the Newcastle Ottawa Scores (NOS) adapted for cross-sectional studies and case-control studies [14].

## 3. Results

**Figure 1:**
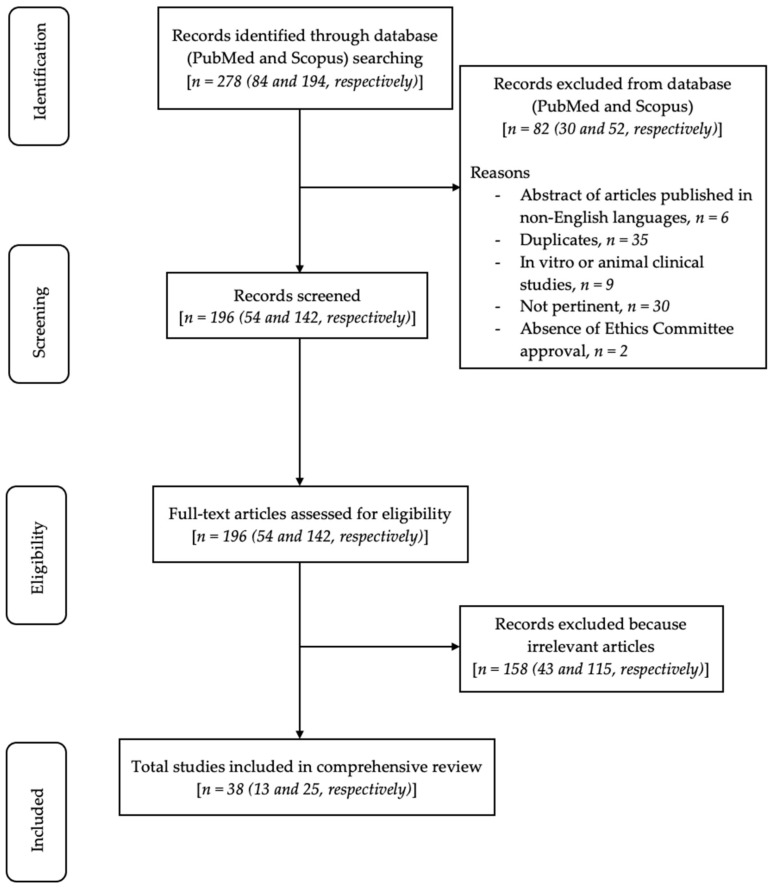
Flow chart of the review process.

The primary search identified 278 articles based on MeSH terms. Following this, 82 articles were removed (6 abstracts of articles published in non-English languages, 35 duplicates, 9 in vitro or animal clinical studies, 30 because they were not pertinent, and 2 because of the absence of Ethics Committee approval), and 196 articles were screened based on title and abstracts. The remaining 196 full-text articles were assessed for eligibility. Additionally, 159 full-text articles were further excluded because they were irrelevant articles. The 37 relevant articles were finally included and analyzed in this review. The flow chart of the review process is described in Figure 1. 

The studies were from four categories: before-after (pre-post) studies with no control group [15,16,17,18,19], observational cohort and cross-sectional studies [20,21,22,23,24,25,26,27,28,29,30,31,32,33,34,35,36,37,38,39,40,41], systematic reviews and meta-analyses [42,43,44,45,46,47], and non-randomized studies [48,49,50,51,52].

### Risk of Bias

The Cochrane Collaboration’s tool for assessing risk of bias was used to evaluate the reviewed articles (Table 1). Table 2 shows the criteria for judging risk of bias in the “risk of bias” assessment tool. This review has a moderate risk of bias.

NIH Quality Assessment Tool for Before-After (Pre-Post) Studies with No Control Group, for Observational Cohort and Cross-Sectional Studies and for Systematic Reviews and Meta-Analyses are shown in Tables S1–S3 (Supplementary Materials), respectively. Quality Assessment of non-randomized studies is shown in Table S4 (Supplementary Materials). Quality analysis through Newcastle Ottawa Scores adapted for the cross-sectional studies and for case-control studies is shown in Tables S5 and S6 (Supplementary Materials).

## 4. Discussion

Major and minor salivary glands biopsy involve the removal of a variable amount of glandular parenchyma, affected by local or systemic inflammatory processes, through a surgical-operative approach defined as an open incisional biopsy; excisional biopsy involves removal of the whole lesion, including a variable amount of bordering glandular tissue [44]. In the SS, characterized by an immune-mediated inflammatory process, it is essential to perform an incisional biopsy of the salivary glands that can express histo-cyto-pathological features to confirm the presence of the pathology. Submandibular glands clearly express pathognomonic changes of SS, yet there are no incisional biopsy techniques, probably because surgical procedures are too invasive and general anesthesia would be required [5].

Two techniques of incisional biopsy of the sublingual glands are reported. Pennec’s technique provides a linear incision from the oral floor mucosa to the adherent gingival mucosa, from the first lower premolar to the lower lateral incisor, without reporting complications. The diagnostic sensitivity and specificity are found to be 72% and 52%, respectively [15]. Adam’s technique suggests a lateral incision to the Wharton’s duct, from the retro-incisive papilla, extending about one centimeter posteriorly, presenting the risk of possible transient post-surgical swelling of the oral floor. No information regarding diagnostic accuracy is reported [20]. A variation to this technique has been proposed by Baurmash and Worth, performing a small Y-shaped superior concavity incision surrounding the auricular lobe inferiorly, without reporting data regarding diagnostic sensitivity and specificity [21]. Complications associated with parotid biopsy techniques, such as transient alteration of pre-auricular surface sensitivity, are uncommon [50]. Palate biopsy can be performed following the technique proposed by Eisenbud, which involves an incision approximately 1 cm from the midline, near the mesial aspect of the second molar, at the border between the hard and soft palate, using a punch biopsy approximately 5 mm in diameter. This technique could result in a postoperative hemorrhagic complication. No information regarding diagnostic sensitivity and specificity accuracy is reported [48].

Finally, several techniques have been suggested for MSGB, which have in common an interest in the glandular units of the lower labial mucosa. The aims to be achieved should include minimal surgical trauma, incisions as small and superficial as possible to determine minimum bleeding, minimize surgical injuries to peripheral nerve endings, reducing the hypo-sensitivity events to external and internal labial surfaces, collect a sufficient volume of glandular structures and, finally, reduce local postoperative complications, especially mid- and long-term paresthesia [32].

In chronological order, the first technique described was by Chisholm and Mason in 1968, proposing a 3 × 1 cm elliptical incision reaching the muscular layer of the lower lip. This technique has been applied to 40 patients, and no major postoperative complications or information regarding its diagnostic accuracy has been reported [16]. In 1975, Greenspan proposed a linear incision of approximately 1.5–2 cm on the lower labial mucosa, parallel to the vermilion border and lateral to the midline. This technique was applied to 75 cases and only 1 case presented a 1 cm^2^ area of localized anesthesia of the lower lip for several months. No information regarding diagnostic sensitivity and specificity is reported [17]. Daniels proposed a single 2 cm horizontal incision parallel to the edge of the vermilion in the center of the lower lip, between the midline and the corner of the lower lip [22]. This technique was applied in 496 patients (3 cases reported labio-sensory complications) with a sensitivity of 82.4% and specificity of 86.2% [22,23]. In 1985, Fox considered the use of mid-palpebral calazio forceps to circumscribe the labial incision area, without reporting information regarding the number of cases with postoperative complications and sensitivity and specificity [24]. In 1988, Marx proposed Greenspan’s technique again, suggesting a 3 × 0.75 cm incision. This technique was applied to 77 cases, reporting 3 cases of partial loss of lip sensitivity (one of which persisted for more than 2 years) and no information regarding diagnostic accuracy [25]. One year later, Delgado and Mosqueda suggested a 10 mm longitudinal incision on the labial mucosa, anterior to the inferior canine. This technique has been applied to 19 cases, no postoperative complications have been reported, and there is no information regarding its diagnostic accuracy [49]. In 1992, Richards proposed a single linear horizontal incision of the mucosal tissue of approximately 1 cm. This technique was applied to 58 cases, of which 2 showed reduced sensitivity in an area ipsilateral to the surgical incision, and reporting sufficient sensitivity and specificity to diagnose most cases of SS, without reporting the percentages [18]. In 2000, Seoane used calazio forceps, performing an elliptical horizontal incision of 1 cm x 4 mm, without reporting information regarding number of cases, possible postoperative complications, and sensitivity and specificity data [26]. In the following year, Peloro described the X-marks technique, i.e., once the salivary glands’ papules are highlighted with a surgical pen, a superficial incision of the labial mucosa of 1.5–2 mm is performed and, finally, a second incision is made perpendicular to the first one. There are no data on number of cases, possible postoperative complications, and diagnostic accuracy [27]. In the same year, Guevara-Gutierrez et al. suggested the punch biopsy technique, using a 4 mm diameter punch scalpel, lightly penetrating the epithelium of the lower lip, between the midline and the labial commissure. This technique was applied to 50 cases (of which 2 cases manifested transient lip numbness) with a sensitivity of 82.4% and specificity of 86.2% [50]. In 2007, Teppo and Revonta described 2–3 mm horizontal micro-incisions, shelling the glands to the surface and gently removing them with scissors and surgical forceps. This technique was applied to 191 cases, of which 1 case presented with pyogenic granuloma of the biopsy wound, and in 82% of patients the biopsy result and subsequent clinical diagnosis were correlated [28]. Finally, in 2020, Comini et al. proposed a minimally invasive technique, using a sharp-tipped needle to pull out the minor salivary glands and remove them, reducing invasiveness and local trauma, representing one of the most recommended approaches for minimally invasive excision of the lower lip glands. This technique was performed in 569 patients without any complications and with an accuracy of 97.6% [29].

The literature review shows a high heterogeneity of MSGB techniques used; in fact, most authors described different techniques according to their clinical and experimental experience. In addition, the sensitivity and specificity or diagnostic accuracy of the biopsy technique is reported for only a few studies, highlighting that Comini’s technique presents the most satisfactory results in the diagnosis of SS.

A summary table of the main open biopsy techniques of the major and minor salivary glands is shown in Table 3.

It is possible to state that MSGB represents the technique recommended to clinicians for a histo-pathological evaluation, verifying the presence of one of the main classification criteria for the diagnosis of SS, enunciated in 2016 by the American College of Rheumatology/European League Against Rheumatism (ACR/EULAR) [33]. In addition, MSGB allows for histopathological diagnosis of other inflammatory or immune-reactive diseases of salivary glandular parenchyma, thanks to value scales and histo-morphologic criteria proposed by Chisholm and Mason, Greenspan and Daniels, and Tarpley. The diagnostic criteria of Chisholm and Mason, established in 1968, include five grades of glandular tissue impairment (0 to 4), based on the presence of peri-glandular lymphocytic infiltration [19]. Greenspan and Daniels in 1974 proposed a second classification, adding the concept of focus score (FS), defined as the number of lymphocyte foci in a 4 mm^2^ area of glandular tissue, and focal lymphocytic sialadenitis (FLS) when FS > 1 is detected [52]. Finally, in the same year, Tarpley introduced the parameters of possible acinar destruction and peri-ductal and peri-acinar connective fibrosis [34].

Frequently, in oncology, US-FNAB represents a useful method to perform a cytopathological diagnosis of likely or confirmed neoplastic lesions, usually following radiographs, which are difficult to investigate with traditional biopsy techniques [42]. The greater rapidity and ease of execution, less painful perception, and high repeatability represent the most obvious advantages of US-FNAB compared with incisional biopsy, improving diagnostic accuracy [34]. It can also be performed during pregnancy, there are no contraindications, and anesthesia is not necessary because it would alter the sensitivity to palpation, reducing the accuracy of injection, it is also minimally invasive, quick to perform, and well tolerated by the patient [36]. The patient may experience occasional pain or discomfort in the postoperative period, rarely a subcutaneous hematoma may appear, and exceptionally a hemorrhagic effusion, organ damage, or surgical wound infection [30].

US-FNAB categorizes most benign (95%) and malignant (93%) salivary gland neoplasms, reducing its accuracy to 73% when the specimen is non-neoplastic [37]. However, a recent systematic review of the literature found that the cytologic diagnosis performed on specimens obtained by parotid US-FNAB was uncertain in 8% of cases, increasing the probability of malignancy at final diagnosis by almost twofold, due to the absence of a standardized terminology system and unambiguous classification for salivary neoplasms [42]. The first categorization of cytologic diagnoses performed by needle aspiration was performed by Fundakowski et al. [38]. Subsequently, following its modification, the Temple (Philadelphia, PA, USA) classification system and the Milan system were developed, improving inter- and intra-institutional reliability [45]. Temple’s system stratifies the risk of occurrence of neoplastic processes into six categories, whereas Milan’s system not only stratifies the risk of occurrence of neoplasms into six categories, but also associates the need for treatment [39,45]. These systems improved clinical decision-making and preoperative counselling [45].

Since the cytological diagnosis of needle aspiration specimens has reduced accuracy for non-neoplastic specimens, US-guided CNB has been introduced as an alternative diagnostic method in the preoperative evaluation of salivary gland lesions due to its high sensitivity and specificity [42]. US-guided CNB is a method that allows collecting tissue fragments, including both the peripheral and central portion of the lesion, under an ultrasound guide, allowing the collection of a greater amount of cytological material than needle aspiration, preserving more of the histological architecture of the specimen, evaluating the extracapsular tumor invasion, and more precisely classifying the lesion [43,46]. After harvesting, the material is fixed in 10% formalin, placed in a paraffin block, 4–5 μm thick sections are made, and finally stained with various reagents to evaluate the architecture, cell morphology, and relationships of the lesion with the surrounding healthy glandular tissue [42,43]. Immunohistochemical staining appears to be more reliable in samples obtained by percutaneous ultrasound-guided biopsy [46]. Finally, the misdiagnosis rate for US-guided CNB is 1.6%, compared with 8% for US-FNAB [40].

Studies have shown that the main complication appears to be the appearance of a hematoma, and the risk of tumor spread is minimal [40]. However, there are some disadvantages associated with its performance: it is more painful than US-FNAB and, consequently, local anesthesia is required; additionally, since it removes more tissue, if it is improperly performed it may result in greater postoperative morbidity [47]. 

IOFSs represent an additional biopsy method to improve the diagnostic underperformance of US-FNAB, whose diagnostic outcome will guide the therapeutic strategy [47]. To quickly highlight the nature of the lesion and the completeness of the surgical excision, the anatomical piece is taken during surgery, subsequently placed in a special gel, frozen at -35°C, sections of few μm thickness are made, laid on a slide, and, finally, stained and analyzed under a microscope [30]. The sensitivity and specificity of IOFSs were found to be 80–90% and 98–99%, respectively, according to the study considered [31,41]. Complications are comparable to those discussed in relation to incisional salivary gland biopsy [47].

This study could be helpful to clinicians as it is an overview as well as a comprehensive and exhaustive guide of the indications and main biopsy techniques of the salivary glands, comparing their complications so that clinicians can independently choose the open surgical technique. In addition, in the presence of salivary gland tumors, it is important for clinicians to be familiar with the most appropriate biopsy techniques in making a prior or intraoperative diagnosis: US-FNAB, US-guided CNB, or IOFSs.

## 5. Conclusions

From this review it is possible to conclude:

- MSGB must be considered a surgical procedure.
- It is better to adopt the technique with the lowest number of reported complications.
- MSGB is important for clinicians in the diagnosis of SS because it represents the only surgical procedure present in the current classification criteria of SS proposed by the ACR/EULAR.
- MSGB allows the diagnosis of inflammatory or immune-mediated diseases thanks to the anatomo-pathological classification systems.
- There still needs to be a consensus in the diagnostic evaluation of samples obtained by MSGB, including additional descriptions such as mean area of focus, area of lymphocyte infiltration, and its degree of organization.
- US-guided CNB could represent a valid diagnostic alternative in relation to the low accuracy present for non-neoplastic lesions and the high percentage of undetermined diagnosis related to US-FNAB.
- IOFSs can be reserved when US-guided CNB is not available and to quickly reveal the nature of the lesion and the completeness of surgical excision.

## Figures and Tables

**Table 1:** Risk of bias of studies is represented by the green symbol, low risk of bias, and the yellow symbol, high risk of bias.

	Random Sequence Generation	Allocation Concealment	Blinding	Incomplete Outcome Data	Selective Reporting
Pennec et al., 1990 [15]	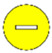	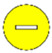	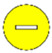	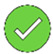	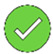
Chisholm et al., 1968 [16]	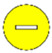	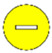	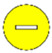	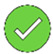	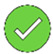
Greenspan et al., 1974 [17]	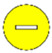	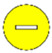	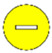	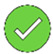	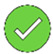
Richards et al., 1992 [18]	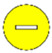	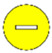	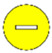	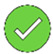	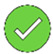
Pan et al., 2020 [19]	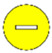	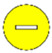	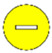	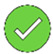	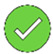
Berquin et al., 2006 [20]	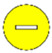	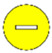	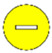	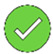	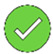
Baurmash et al., 2005 [21]	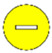	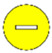	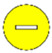	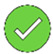	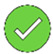
Daniels et al., 1984 [22]	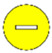	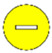	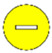	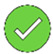	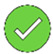
Vitali et al., 1994 [23]	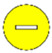	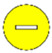	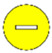	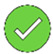	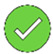
Fox et al., 1985 [24]	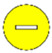	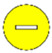	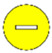	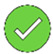	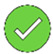
Marx et al., 1988 [25]	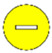	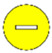	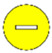	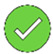	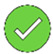
Seoane et al., 2000 [26]	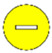	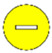	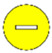	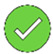	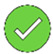
Peloro et al., 2001 [27]	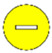	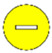	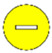	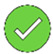	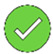
Teppo et al., 2007 [28]	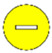	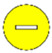	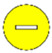	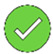	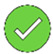
Comini et al., 2020 [29]	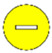	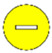	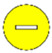	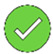	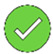
Liao et al., 2017 [30]	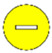	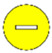	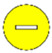	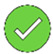	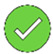
Pastorello et al., 2021 [31]	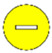	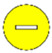	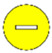	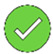	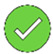
Wijaja et al., 2019 [32]	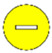	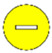	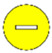	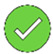	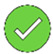
Shiboski et al., 2017 [33]	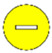	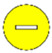	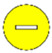	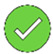	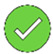
Manzo C, 2019 [34]	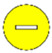	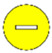	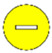	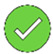	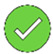
Zbären et al., 2018 [35]	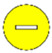	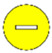	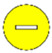	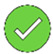	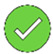
Varoni et al., 2020 [36]	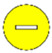	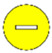	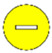	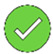	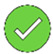
Strieder et al., 2022 [37]	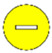	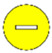	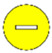	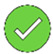	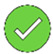
Fundakowski et al., 2014 [38]	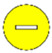	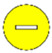	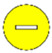	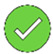	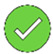
Kaushik et al., 2020 [39]	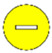	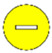	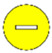	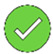	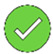
Walsh et al., 2022 [40]	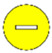	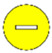	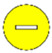	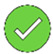	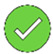
Suzuki et al., 2019 [41]	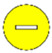	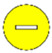	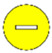	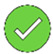	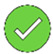
Cho et al., 2020 [42]	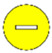	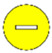	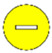	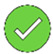	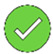
Hurry et al., 2022 [43]	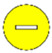	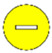	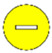	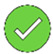	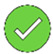
Pontarini et al., 2021 [44]	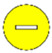	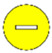	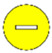	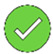	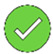
Luo et al., 2019 [45]	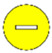	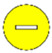	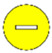	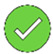	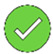
Kim et al., 2018 [46]	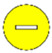	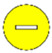	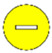	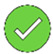	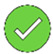
Howlett et al., 2016 [47]	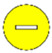	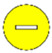	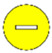	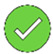	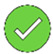
Eisenbud et al., 1973 [48]	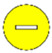	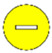	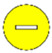	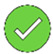	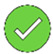
Delgado et al., 1989 [49]	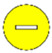	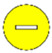	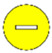	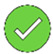	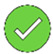
Guevara-Gutiérrez et al., 2001 [50]	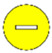	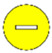	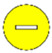	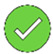	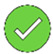
Pijpe et al., 2007 [51]	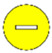	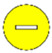	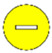	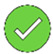	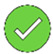
Tsukamoto et al., 2020 [52]	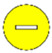	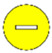	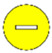	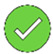	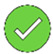

**Table 2:** Criteria for judging risk of bias in the “Risk of bias” assessment tool.

**Random Sequence Generation**	
Criteria for a judgement of ‘Low risk’ of bias.	The investigators describe a random component in the sequence generation process.
Criteria for the judgement of ‘High risk’ of bias.	The investigators describe a non-random component in the sequence generation process. Usually, the description would involve some systematic, non-random approach. Other non-random approaches happen much less frequently than the systematic approaches mentioned above and tend to be obvious. They usually involve judgement or some method of non-random categorization of participants.
**Allocation Concealment**	
Criteria for a judgement of ‘Low risk’ of bias.	Participants and investigators enrolling participants could not foresee assignment because one of the following, or an equivalent method, was used to conceal allocation.
Criteria for the judgement of ‘High risk’ of bias.	Participants or investigators enrolling participants could possibly foresee assignments and thus introduce selection bias.
**Blinding**	
Criteria for a judgement of ‘Low risk’ of bias.	Any one of the following: - No blinding or incomplete blinding, but the review authors judge that the outcome is not likely to be influenced by lack of blinding; - Blinding of participants and key study personnel ensured, and unlikely that the blinding could have been broken; - No blinding of outcome assessment, but the review authors judge that the outcome measurement is not likely to be influenced by lack of blinding; - Blinding of outcome assessment ensured, and unlikely that the blinding could have been broken.
Criteria for the judgement of ‘High risk’ of bias.	Any one of the following: - No blinding or incomplete blinding, and the outcome is likely to be influenced by lack of blinding; - Blinding of key study participants and personnel attempted, but likely that the blinding could have been broken, and the outcome is likely to be influenced by lack of blinding; - No blinding of outcome assessment, and the outcome measurement is likely to be influenced by lack of blinding; - Blinding of outcome assessment, but likely that the blinding could have been broken, and the outcome measurement is likely to be influenced by lack of blinding.
**Incomplete Outcome Data**	
Criteria for a judgement of ‘Low risk’ of bias.	Any one of the following: - No missing outcome data; - Reasons for missing outcome data unlikely to be related to true outcome (for survival data, censoring unlikely to be introducing bias); - Missing outcome data balanced in numbers across intervention groups, with similar reasons for missing data across groups; - For dichotomous outcome data, the proportion of missing outcomes compared with observed event risk not enough to have a clinically relevant impact on the intervention effect estimate; - For continuous outcome data, plausible effect size (difference in means or standardized difference in means) among missing outcomes not enough to have a clinically relevant impact on observed effect size; - Missing data have been imputed using appropriate methods.
Criteria for the judgement of ‘High risk’ of bias.	Any one of the following: - Reason for missing outcome data likely to be related to true outcome, with either imbalance in numbers or reasons for missing data across intervention groups; - For dichotomous outcome data, the proportion of missing outcomes compared with observed event risk enough to induce clinically relevant bias in intervention effect estimate; - For continuous outcome data, plausible effect size (difference in means or standardized difference in means) among missing outcomes enough to induce clinically relevant bias in observed effect size; - ‘As-treated’ analysis done with substantial departure of the intervention received from that assigned at randomization; - Potentially inappropriate application of simple imputation.
**Selective Reporting**	
Criteria for a judgement of ‘Low risk’ of bias.	Any one of the following: - The study protocol is available and all of the study’s pre-specified (primary and secondary) outcomes that are of interest in the review have been reported in the pre-specified way; - The study protocol is not available but it is clear that the published reports include all expected outcomes, including those that were pre-specified (convincing text of this nature may be uncommon).
Criteria for the judgement of ‘High risk’ of bias.	Any one of the following: - Not all of the study’s pre-specified primary outcomes have been reported; - One or more primary outcomes is reported using measurements, analysis methods or subsets of the data (e.g., subscales) that were not pre-specified; - One or more reported primary outcomes were not pre-specified (unless clear justification for their reporting is provided, such as an unexpected adverse effect); - One or more outcomes of interest in the review are reported incompletely so that they cannot be entered in a meta-analysis; - The study report fails to include results for a key outcome that would be expected to have been reported for such a study.

**Table 3:** Summary table of the main open biopsy techniques of the major and minor salivary glands.

	**Biopsy Site**	**Biopsy Technique**	**Potential** **Complications**
Pennec et al., 1990 [15]	Sublingual salivary gland	Linear incision from the oral floor mucosa to the adherent gingival mucosa, from the first lower premolar to the lower lateral incisor	Authors report no complications
Chisholm et al., 1968 [16]	Minor salivary glands	3 × 1 cm elliptical incision reaching the muscular layer of the lower lip	Authors report no complications
Greenspan et al., 1974 [17]	Minor salivary glands	Linear incision of approximately 1.5–2 cm on the lower labial mucosa, parallel to the vermilion border and lateral to the midline	Chronic hypoesthesia for several months
Richards et al., 1992 [18]	Minor salivary glands	Single linear horizontal incision of the mucosal tissue of approximately 1 cm	Reduced postsurgical surface sensitivity
Berquin et al., 2006 [20]	Sublingual salivary gland	Lateral incision to the Wharton’s duct, from the retro-incisive papilla, extending about one centimeter posteriorly	Transient post-surgical swelling of the oral floor
Baurmash et al., 2005 [21]	Parotid gland	Small Y-shaped, superior concavity incision surrounding the auricular lobe inferiorly	Transient alteration of pre-auricular surface sensitivity
Daniels et al., 1984 [22]	Minor salivary glands	2 cm horizontal incision parallel to the edge of the vermilion in the center of the lower lip, between the midline and the corner of the lower lip	Labio-sensory complications
Fox et al., 1985 [24]	Minor salivary glands	Circumscription of the labial incision area by a mid-palpebral calazio forceps	No data regarding the number of cases with postoperative complications
Marx et al., 1988 [25]	Minor salivary glands	3 × 0.75 cm elliptical incision reaching the muscular layer of the lower lip	Partial loss of labial sensitivity
Seoane et al., 2000 [26]	Minor salivary glands	Elliptical horizontal incision of 1 cm × 4 mm	No data regarding the number of cases with postoperative complications
Peloro et al., 2001 [27]	Minor salivary glands	X-marks technique: highlight salivary gland papules with a surgical pen, perform a superficial incision of the labial mucosa of 1.5–2 mm and, finally, a second incision perpendicular to the first one	No data regarding the number of cases with postoperative complications
Teppo et al., 2007 [28]	Minor salivary glands	2–3 mm horizontal micro-incisions, shelling the glands came to the surface and gently removing them with scissors and surgical forceps	Pyogenic granuloma of biopsy wound
Comini et al., 2020 [29]	Minor salivary glands	Extraction of the minor salivary glands using a sharp-tipped needle	Authors report no complications
Eisenbud et al., 1973 [48]	Hard palate	Incision approximately 1 cm from the midline, near mesial aspect of the second molar, at the border between the hard and soft palate, using a punch biopsy approximately 5 mm in diameter	Postoperative hemorrhagic complication
Delgado et al., 1989 [49]	Minor salivary glands	10 mm longitudinal incision on the labial mucosa, anterior to the inferior canine	Authors report no complications
Guevara-Gutiérrez et al., 2001 [50]	Minor salivary glands	Punch biopsy technique: lightly penetrate the epithelium of the lower lip using a 4 mm diameter punch scalpel, between the midline and the labial commissure	Modest transient hyposensitivity of the lower lip

## Data Availability

Upon request to the corresponding author, the data are available for use.

## Supplementary Materials

The following supporting information can be downloaded at: https://www.mdpi.com/article/10.3390/healthcare10081537/s1, Table S1: NIH Quality Assessment for Before-After (Pre-Post) Studies With No Control Group; Table S2: NIH Quality Assessment Tool for Observational Cohort and Cross-Sectional Studies; Table S3: NIH Quality Assessment of Systematic Reviews and Meta-Analyses; Table S4: Methodological Index for Non-Randomized Studies (MINORS) criteria; Table S5: Newcastle Ottawa Scores adapted for the cross-sectional studies; Table S6: Newcastle Ottawa Scores for case-control studies.
