# Supplementary material for: Current Salivary Glands Biopsy Techniques: A Comprehensive Review
Source: Healthcare (Basel). 2022 Aug 14;10(8):1537. doi: 10.3390/healthcare10081537 (PMC9408798; doi:10.3390/healthcare10081537)
Supplement: Supplementary file 1 [file healthcare-10-01537-s001.zip › healthcare-1829402-supplementary.pdf]

Table S1. NIH Quality Assessment for Before-After (Pre-Post) Studies With No Control Group.

NIH Quality Assessment Tool for Before-After (Pre-Post) Studies With No Control Group														
**First Author et al., Year**	**Q1**	**Q2**	**Q3**	**Q4**	**Q5**	**Q6**	**Q7**	**Q8**	**Q9**	**Q10**	**Q11**	**Q12**	**Total Score**	**Quality Rating**
Pennec et al., 1990 [1]	Y	Y	Y	N	Y	Y	Y	N	NR	N	N	Y	7/12 (58.33%)	Fair
Chisholm et al., 1968 [2]	Y	Y	Y	Y	NR	Y	Y	N	NR	N	N	Y	7/12 (58.33%)	Fair
Greenspan et al., 1974 [3]	Y	Y	Y	Y	N	Y	Y	NR	Y	N	Y	Y	9/12 (75.00%)	Good
Richards et al., 1992 [4]	Y	Y	Y	Y	Y	Y	Y	N	Y	N	N	Y	9/12 (75.00%)	Good
Pan et al., 2020 [5]	Y	Y	Y	Y	NR	Y	Y	N	NR	N	N	Y	7/12 (58.33%)	Fair

Q1: Was the study question or objective clearly stated?, Q2: Were eligibility/selection criteria for the study population prespecified and clearly described?, Q3: Were the participants in the study representative of those who would be eligible for the test/service/intervention in the general or clinical population of interest?, Q4: Were all eligible participants that met the prespecified entry criteria enrolled?, Q5: Was the sample size sufficiently large to provide confidence in the findings?, Q6: Was the test/service/intervention clearly described and delivered consistently across the study population?, Q7: Were the outcome measures prespecified, clearly defined, valid, reliable, and assessed consistently across all study participants?, Q8: Were the people assessing the outcomes blinded to the participants' exposures/interventions?, Q9: Was the loss to follow-up after baseline 20% or less? Were those lost to follow-up accounted for in the analysis?, Q10: Did the statistical methods examine changes in outcome measures from before to after the intervention? Were statistical tests done that provided p values for the pre-to-post changes?, Q11: Were outcome measures of interest taken multiple times before the intervention and multiple times after the intervention (i.e., did they use an interrupted time-series design)?, Q12: If the intervention was conducted at a group level (e.g., a whole hospital, a community, etc.) did the statistical analysis take into account the use of individual-level data to determine effects at the group level?; Total Score: Number of yes; CD: cannot be determined; NA: not applicable; NR: not reported; N: no; Y: yes. Quality Rating: Poor <50%, Fair 50–75%, Good ≥75%.

Table S2. NIH Quality Assessment Tool for Observational Cohort and Cross-Sectional Studies.

	NIH Quality Assessment Tool for Observational Cohort and Cross-Sectional Studies														
**First Author et al., Year**	**Q1**	**Q2**	**Q3**	**Q4**	**Q5**	**Q6**	**Q7**	**Q8**	**Q9**	**Q10**	**Q11**	**Q12**	**Q13**	**Q14**	**Total Score**	**Quality Rating**
Berquin et al., 2006 [6]	Y	Y	Y	Y	N	N	Y	N	Y	N	Y	N	Y	N	8/14 (57.14%)	Fair
Baurmash et al., 2005 [7]	Y	Y	NR	Y	NR	Y	Y	Y	Y	Y	Y	NR	N	Y	10/14 (71.42%)	Fair
Daniels et al., 1984 [8]	Y	Y	NR	Y	N	Y	Y	NR	NR	NR	Y	NR	Y	N	7/14 (50.00%)	Fair
Vitali et al., 1994 [9]	Y	Y	Y	Y	NR	Y	Y	Y	Y	Y	Y	NR	Y	Y	12/14 (85.71%)	Good
Fox et al., 1985 [10]	Y	Y	N	Y	NR	Y	Y	Y	NR	N	Y	N	NR	N	7/14 (50.00%)	Fair
Marx et al., 1988 [11]	Y	Y	Y	Y	NR	Y	Y	Y	Y	Y	Y	NR	Y	Y	12/14 (85.71%)	Good
Seoane et al., 2000 [12]	Y	Y	NR	Y	N	Y	Y	NR	NR	NR	Y	NR	Y	N	7/14 (50.00%)	Fair
Peloro et al., 2001 [13]	Y	Y	Y	Y	N	N	Y	N	Y	N	Y	N	Y	N	8/14 (57.14%)	Fair
Teppo et al., 2007 [14]	Y	Y	Y	Y	NR	Y	Y	Y	Y	Y	Y	NR	Y	Y	12/14 (85.71%)	Good
Comini et al., 2020 [15]	Y	Y	Y	Y	NR	Y	Y	Y	Y	Y	Y	NR	Y	Y	12/14 (85.71%)	Good
Liao et al., 2017 [16]	Y	Y	Y	Y	NR	Y	Y	Y	Y	NR	Y	N	NR	Y	10/14 (71.42%)	Fair
Pastorello et al., 2021 [17]	Y	Y	Y	Y	NR	Y	Y	Y	Y	NR	Y	N	NR	Y	10/14 (71.42%)	Fair
Wijaja et al., 2019 [18]	Y	Y	Y	Y	NR	Y	Y	Y	Y	Y	Y	NR	Y	Y	12/14 (85.71%)	Good
Shiboski et al., 2017 [19]	Y	Y	Y	Y	NR	Y	Y	Y	Y	Y	Y	NR	Y	Y	12/14 (85.71%)	Good
Manzo C, 2019 [20]	Y	Y	Y	Y	N	N	Y	N	Y	N	Y	N	Y	N	8/14 (57.14%)	Fair

Zbären et al., 2018 [**21**]	Y	Y	NR	Y	NR	Y	Y	Y	Y	Y	Y	NR	N	Y	10/14 (71.42%)	Fair
Varoni et al., 2020 [**22**]	Y	Y	Y	Y	NR	Y	Y	Y	Y	Y	Y	NR	Y	Y	12/14 (85.71%)	Good
Strieder et al., 2022 [**23**]	Y	Y	Y	Y	NR	Y	Y	Y	Y	Y	Y	NR	Y	Y	12/14 (85.71%)	Good
Fundakowski et al., 2014 [**24**]	Y	Y	Y	Y	NR	Y	Y	Y	Y	NR	Y	N	NR	Y	10/14 (71.42%)	Fair
Kaushik et al., 2020 [**25**]	Y	Y	Y	Y	NR	Y	Y	Y	Y	Y	Y	NR	Y	Y	12/14 (85.71%)	Good
Walsh et al., 2022 [**26**]	Y	Y	Y	Y	NR	Y	Y	Y	Y	Y	Y	NR	Y	Y	12/14 (85.71%)	Good
Suzuki et al., 2019 [**27**]	Y	Y	Y	Y	NR	Y	Y	Y	Y	Y	Y	NR	Y	Y	12/14 (85.71%)	Good

Q1: Was the research question or objective in this paper clearly stated?, Q2: Was the study population clearly specified and defined?, Q3: Was the participation rate of eligible persons at least 50%?, Q4: Were all the subjects selected or recruited from the same or similar populations (including the same time period)? Were inclusion and exclusion criteria for being in the study prespecified and applied uniformly to all participants?, Q5: Was a sample size justification, power description, or variance and effect estimates provided?, Q6: For the analyses in this paper, were the exposure(s) of interest measured prior to the outcome(s) being measured?, Q7: Was the timeframe sufficient so that one could reasonably expect to see an association between exposure and outcome if it existed?, Q8: For exposures that can vary in amount or level, did the study examine different levels of the exposure as related to the outcome (e.g., categories of exposure, or exposure measured as continuous variable)?, Q9: Were the exposure measures (independent variables) clearly defined, valid, reliable, and implemented consistently across all study participants?, Q10: Was the exposure(s) assessed more than once over time?, Q11: Were the outcome measures (dependent variables) clearly defined, valid, reliable, and implemented consistently across all study participants?, Q12: Were the outcome assessors blinded to the exposure status of participants?, Q13: Was loss to follow-up after baseline 20% or less?, Q14: Were key potential confounding variables measured and adjusted statistically for their impact on the relationship between exposure(s) and outcome(s)?; Total Score: Number of yes; CD: cannot be determined; NA: not applicable; NR: not reported; N: no; Y: yes. Quality Rating: Poor <50%, Fair 50 – 75%, Good ⩾75%.

Table S3. NIH Quality Assessment of Systematic Reviews and Meta-Analyses.

	NIH Quality Assessment of Systematic Reviews and Meta-Analyses									
**First Author et al., Year**	**Q1**	**Q2**	**Q3**	**Q4**	**Q5**	**Q6**	**Q7**	**Q8**	**Total Score**	**Quality Rating**
Cho et al., 2020 [**28**]	NR	Y	Y	Y	Y	Y	Y	Y	7/8 (87.5%)	Good
Hurry et al., 2022 [**29**]	Y	Y	Y	Y	NR	Y	NR	Y	6/8 (75.00%)	Good
Pontarini et al., 2021 [**30**]	Y	Y	Y	Y	NR	Y	NR	Y	6/8 (75.00%)	Good
Luo et al., 2019 [**31**]	Y	Y	Y	Y	NR	Y	Y	Y	7/8 (87.5%)	Good
Kim et al., 2018 [**32**]	NR	Y	Y	Y	Y	Y	Y	Y	7/8 (87.5%)	Good
Howlett et al., 2016 [**33**]	Y	Y	Y	Y	NR	Y	NR	Y	6/8 (75.00%)	Good

Q1: Is the review based on a focused question that is adequately formulated and described?, Q2: Were eligibility criteria for included and excluded studies predefined and specified?, Q3: Did the literature search strategy use a comprehensive, systematic approach?, Q4: Were titles, abstracts, and full-text articles dually and independently reviewed for inclusion and exclusion to minimize bias?, Q5: Was the quality of each included study rated independently by two or more reviewers using a standard method to appraise its internal validity?, Q6: Were the included studies listed along with important characteristics and results of each study?, Q7: Was publication bias assessed?, Q8: Was heterogeneity assessed? (This question applies only to meta-analyses.)?, Total Score: Number of yes; CD: cannot be determined; NA: not applicable; NR: not reported; N: no; Y: yes. Quality Rating: Poor <50%, Fair 50 – 75%, Good ⩾75%.

Table S4. Methodological Index for Non-Randomized Studies (MINORS) criteria.

	Methodological Index for Non-Randomized Studies (MINORS) criteria													
**First Author et al., Year**	**Q1**	**Q2**	**Q3**	**Q4**	**Q5**	**Q6**	**Q7**	**Q8**	**Q9**	**Q10**	**Q11**	**Q12**	**Total Score**	**Quality Rating**
Eisenbud et al., 1973 [**34**]	2	2	1	2	0	0	0	0	2	2	2	2	15	B
Delgado et al., 1989 [**35**]	2	1	2	2	2	0	0	0	2	2	2	2	17	A
Guevara-Gutiérrez et al., 2001 [**36**]	2	2	2	2	0	2	2	0	2	2	2	2	20	A
Pijpe et al., 2007 [**37**]	2	2	2	2	0	2	2	0	2	2	2	2	20	A
Tsukamoto et al., 2020 [**38**]	2	2	2	2	0	2	2	0	2	2	2	2	20	A

Q1: A clearly stated aim, Q2: Inclusion of consecutive patients, Q3: Prospective collection of data, Q4: Endpoints appropriate to the aim of the study, Q5: Unbiased assessment of the study endpoint, Q6: Follow-up period appropriate to the aim of the study, Q7: Loss to follow up less than 5%, Q8: Prospective calculation of the study size, Q9: An adequate control group, Q10: Contemporary groups, Q11: Baseline equivalence of groups, Q12: Adequate statistical analyses; Total Score: sum of item score; 0: not reported; 1: reported but inadequate, 2: reported and adequate . Quality Rating: A, 16 or 24, low risk of bias; B, >12-<16 or >20-<24, low risk of bias; C, ≤12 or ≤20, high risk of bias.

Table S5. Newcastle Ottawa Scores adapted for the cross-sectional studies.

Newcastle Ottawa Scores adapted for the cross-sectional studies									
**First Author et al., Year**	**Q1**	**Q2**	**Q3**	**Q4**	**Q5**	**Q6**	**Q7**	**Total Score**	**Quality Rating**
Berquin et al., 2006 [6]	1	0	0	1	2	2	1	7	Good
Baurmash et al., 2005 [7]	0	0	0	1	2	2	0	5	Satisfactory
Daniels et al., 1984 [8]	0	0	0	1	2	2	0	5	Satisfactory
Vitali et al., 1994 [9]	1	0	1	1	2	2	1	8	Good
Fox et al., 1985 [10]	0	0	0	1	2	2	0	5	Satisfactory
Marx et al., 1988 [11]	1	0	1	2	2	2	1	9	Very Good
Seoane et al., 2000 [12]	0	0	0	1	2	2	0	5	Satisfactory
Peloro et al., 2001 [13]	0	0	0	1	2	2	0	5	Satisfactory
Teppo et al., 2007 [14]	1	0	1	1	2	2	1	8	Good
Comini et al., 2020 [15]	1	0	1	1	2	2	1	8	Good
Liao et al., 2017 [16]	1	0	1	2	2	2	1	9	Very Good
Pastorello et al., 2021 [17]	1	0	1	1	2	2	1	8	Good
Wijaja et al., 2019 [18]	1	0	1	2	2	2	1	9	Very Good
Shiboski et al., 2017 [19]	1	0	1	2	2	2	1	9	Very Good
Manzo C, 2019 [20]	0	0	0	1	2	2	0	5	Satisfactory

Zbären et al., 2018 [**21**]	0	0	0	1	2	2	0	5	Satisfactory
Varoni et al., 2020 [**22**]	1	0	1	1	2	2	1	8	Good
Strieder et al., 2022 [**23**]	1	0	1	1	2	2	1	8	Good
Fundakowski et al., 2014 [**24**]	0	0	0	1	2	2	0	5	Satisfactory
Kaushik et al., 2020 [**25**]	1	0	1	2	2	2	1	9	Very Good
Walsh et al., 2022 [**26**]	1	0	1	2	2	2	1	9	Very Good
Suzuki et al., 2019 [**27**]	1	0	1	2	2	2	1	9	Very Good

Q1: Representativeness of the sample, Q2: Sample size, Q3: Non-respondents, Q4: Ascertainment of the exposure (risk factor), Q5: Comparability of subjects in different outcome groups on the basis of design or analysis. Confounding factors controlled, Q6: Assessment of outcome, Q7: Statistical test, Total Score: sum of item score points. Quality Rating: 9-10 points, very good studies; 7-8 points, good studies; 5-6 points, satisfactory studies; 0-4 points, unsatisfactory studies.

Table S6. Newcastle Ottawa Scores for case-control studies.

	Newcastle Ottawa Scores for case-control studies									
**First Author et al., Year**	**Q1**	**Q2**	**Q3**	**Q4**	**Q5**	**Q6**	**Q7**	**Q8**	**Total Score**	**Quality Rating**
Eisenbud et al., 1973 [34]	1	0	1	0	0	1	1	1	5	Good
Delgado et al., 1989 [35]	1	1	1	0	1	1	1	1	7	Very Good
Guevara-Gutiérrez et al., 2001 [36]	1	0	1	1	1	1	1	1	7	Very Good
Pijpe et al., 2007 [37]	1	1	1	0	1	1	1	1	7	Very Good
Tsukamoto et al., 2020 [38]	1	1	1	0	1	1	1	1	7	Very Good

Q1: Is the case definition adequate Q2: Representativeness of the cases, Q3: Selection of Controls, Q4: Definition of Controls, Q5: Comparability of cases and controls on the basis of the design or analysis, Q6: Ascertainment of exposure, Q7: Same method of ascertainment for cases and controls, Q8: Non-Response rate, Total Score: sum of item score points. Quality Rating: 7-8 points, very good studies; 5-6 points, good studies; 3-4 points, satisfactory studies; 0-2 points, unsatisfactory studies.

References

1. Pennec, Y.L.; Leroy, J.P.; Jouquan, J.; Lelong, A.; Katsikis, P.; Youinou, P. Comparison of labial and sublingual salivary gland biopsies in the diagnosis of Sjogren's syndrome. *Ann Rheum Dis.* **1990**, *49*, 37–39.
2. Chisholm, D.M.; Mason, D.K. Labial salivary gland biopsy in Sjogren's disease. *J Clin Pathol.* **1968**, *21*, 656–660.
3. Greenspan, J.S.; Daniels, T.E.; Talal, N.; Sylvester, R.A. The histopathology of Sjögren's syndrome in labial salivary gland biopsies. *Oral Surg Oral Med Oral Pathol.* **1974**, *37*, 217–229.
4. Richards, A.; Mutlu, S.; Scully, C.; Maddison, P. Complications associated with labial salivary gland biopsy in the investigation of connective tissue disorders. *Ann Rheum Dis.* **1992**, *51*, 996–997.
5. Pan, X.; Huang, F.; Pan, Z.; Tian, M. Treatment of serologically negative Sjögren's syndrome with tacrolimus: A case report. *J Int Med Res.* **2020**, *48*, 300060519893838.
6. Berquin, K.; Mahy, P.; Weynand, B.; Reychler, H. Accessory or sublingual salivary gland biopsy to assess systemic disease: a comparative retrospective study. *Eur Arch Otorhinolaryngol.* **2006**, *263*, 233–236.
7. Baurmash, H. Parotid biopsy technique. *J Oral Maxillofac Surg.* **2005**, *63*, 1556–1557.
8. Daniels, T.E. Labial salivary gland biopsy in sjögren's syndrome. *Arthritis Rheum.* **1984**, *27*, 147–56.
9. Vitali, C.; Moutsopoulos, H.M.; Bombardieri, S. The European Community Study Group on diagnostic criteria for Sjögren's syndrome. Sensitivity and specificity of tests for ocular and oral involvement in Sjögren's syndrome. *Ann Rheum Dis.* **1994**, *53*, 637-647.
10. Fox, P.C. Simplified Biopsy Technique for Labial Minor Salivary Glands. *Plast Reconstr Surg.* **1985**, *75*, 592–593.
11. Marx, R.E.; Hartman, K.S.; Rethman, K.V. A prospective study comparing incisional labial to incisional parotid biopsies in the detection and confirmation of sarcoidosis, Sjögren's disease, sialosis and lymphoma. *J Rheumatol.* **1988**, *15*, 621–629.
12. Seoane, J.; Varela-Centelles, P.I.; Diz-Dios, P.; Romero, M. Use of Chalazion Forceps to Ease Biopsy of Minor Salivary Glands. *Laryngoscope.* **2000**, *110*, 486–487.
13. Peloro, T.M.; Ramsey, M.L.; Marks, V.J. Surgical Pearl: "X" marks the spot for the salivary gland biopsy. *J Am Acad Dermatol.* **2001**, *45*, 122–123.
14. Teppo, H.; Revonta, M. A follow-up study of minimally invasive lip biopsy in the diagnosis of Sjögren's syndrome. *Clin Rheumatol.* **2007**, *26*, 1099–1103.
15. Comini, L.V.; Lazio, M.S.; Taverna, C.; Luparello, P.; et al. A new, easy, and swift technique for minor salivary gland biopsy in Sjogren's syndrome. *Laryngoscope.* **2020**, *130*, 873–875.
16. Liao, L.J.; Lo, W.-C.; Hsu, W.-L.; Cheng, P.-W.; Wang, C.-P. Assessment of pain score and specimen adequacy for ultrasound-guided fine-needle aspiration biopsy of thyroid nodules. *J Pain Res.* **2017**, *11*, 61–66.
17. Pastorello, R.G.; Rodriguez, E.F.; McCormick, B.A.; Calsavara, V.F.; Chen, L.C.; Zarka, M.A.; et al. Is there a Role for Frozen Section Evaluation of Parotid Masses After Preoperative Cytology or Biopsy Diagnosis?. *Head Neck Pathol.* **2021**, *15*, 859–865.
18. Wijaya, C.; Ramli, R.R.; Khoo, S.G. Dry surgical field minor salivary gland harvest using a chalazion clamp for sicca syndrome. *J Laryngol Otol.* **2019**, *133*, 419–423.
19. Shiboski, C.H.; Shiboski, S.C.; Seror, R.; Criswell, L.A.; et al. 2016 American College of Rheumatology/European League Against Rheumatism Classification Criteria for Primary Sjögren's Syndrome: A Consensus and Data-Driven Methodology Involving Three International Patient Cohorts. *Arthritis Rheumatol.* **2017**, *69*, 35–45.
20. Manzo, C. Labial salivary gland biopsy and secondary Sjögren's syndrome: where we are and where we want to be. *Reumatologia.* **2019**, *57*, 354–355.
21. Zbären, P.; Triantafyllou, A.; Devaney, K.O.; Poorten, V.V.; Hellquist, H.; Rinaldo, A.; et al. Preoperative diagnostic of parotid gland neoplasms: fine-needle aspiration cytology or core needle biopsy?. *Eur Arch Otorhinolaryngol.* **2018**, *275*, 2609–2613.
22. Varoni, E.M.; Villani, G.; Lombardi, N.; Pispero, A.; Lodi, G.; Sardella, A.; et al. Local complications associated with labial salivary gland biopsy for diagnosis of Sjögren's Syndrome: A retrospective cohort study. *J Clin Exp Dent.* **2020**, *12*, e713–e718.
23. Strieder, D.L.; Cristo, A.P.; Zanella, A.B.; Faccin, C.S.; Farenzena, M.; Graudenz, M.C.; et al. Using an ultrasonography risk stratification system to enhance the thyroid fine needle aspiration performance. *Eur J Radiol.* **2022**, *150*, 110244.
24. Fundakowski, C.; Castaño, J.; Abouyared, M.; Lo, K.; Rivera, A.; Ojo, R.; et al. The role of indeterminate fine-needle biopsy in the diagnosis of parotid malignancy. *Laryngoscope.* **2014**, *124*, 678–681.

25. Kaushik, R.; Bhatia, K.; Sarin, H.; Gautam, D.; Sarin, D. Incorporation of the Milan system in reporting salivary gland fine needle aspiration cytology–An insight into its value addition to the conventional system. *Diagn Cytopathol.* **2020**, *48*, 17–29.
26. Walsh, E.; Allan, K.; Brennan, P.A.; Tullett, M.; Gomez, R.S.; Rahimi, S. Diagnostic accuracy of ultrasonography-guided core needle biopsy of parotid gland neoplasms: A large, single-institution experience in United Kingdom. *J Oral Pathol Med.* **2022**, *51*, 1–4.
27. Suzuki, M.; Nakaegawa, Y.; Kobayashi, T.; Kawase, T.; Matsuzuka, T.; Murono, S. The role of frozen section biopsy for parotid gland tumour with benign fine-needle aspiration cytology. *J Laryngol Otol.* **2019**, *133*, 227–229.
28. Cho, J.; Kim, J.; Lee, J.S.; Chee, C.G.; Kim, Y.; Choi, S.I. Comparison of core needle biopsy and fine-needle aspiration in diagnosis of malignant salivary gland neoplasm: Systematic review and meta-analysis. *Head Neck.* **2020**, *42*, 3041–3050.
29. Hurry, K.J.; Karunaratne, D.; Westley, S.; Booth, A.; Ramesar, K.C.R.B.; Zhang, T.T.; Williams, M:; Howlett, D.C. Ultrasound-guided core biopsy in the diagnosis of parotid neoplasia: an overview and update with a review of the literature. *Br J Radiol.* **2022**, *95*, 20210972.
30. Pontarini, E.; Coleby, R.; Bombardieri, M. Cellular and molecular diversity in Sjogren's syndrome salivary glands: Towards a better definition of disease subsets. *Semin Immunol.* **2021**, 101547.
31. Luo, X,; Jhala, N.; Khurana, J.S.; Fundakowski, C.; Jhala, D.N.; Wang, H. Moving Toward a Systematic Approach for Reporting Salivary Gland Cytopathology: An Institutional Experience and Literature Review. *Arch Pathol Lab Med.* **2019**, *143*, 664–669.
32. Kim, H.J.; Kim, J.S. Ultrasound-guided core needle biopsy in salivary glands: A meta-analysis. *Laryngoscope.* **2018**, *128*, 118–125.
33. Howlett, D.C.; Triantafyllou, A. Evaluation: Fine Needle Aspiration Cytology, Ultrasound-Guided Core Biopsy and Open Biopsy Techniques. *Adv Otorhinolaryngol.* **2016**, *78*, 39–45.
34. Eisenbud, L.; Platt, N.; Stern, M.; D'Angelo, W.; Sumner, P. Palatal biopsy as a diagnostic aid in the study of connective tissue diseases. *Oral Surg Oral Med Oral Pathol.* **1973**, *35*, 642–648.
35. Delgado, W.A.; Mosqueda, A. A highly sensitive method for diagnosis of secondary amyloidosis by labial salivary gland biopsy. *J Oral Pathol Med.* **1989**, *18*, 310–314.
36. Guevara-Gutiérrez, E.; Tlacuilo-Parra, A.; Minjares-Padilla, L.M. Minor Salivary Gland Punch Biopsy for Evaluation of Sjögren's Syndrome. *J Clin Rheumatol.* **2001**, *7*, 401–402.
37. Pijpe, J.; Kalk, W.W.I.; van der Wal, J.E.; Vissink, A.; Kluin, P.M.; Roodenburg, J.L.N.; et al. Parotid gland biopsy compared with labial biopsy in the diagnosis of patients with primary Sjogren's syndrome. *Rheumatology (Oxford).* **2006**, *46*, 335–341.
38. Tsukamoto, M.; Suzuki, K.; Tsunoda, K.; Ikeura, K.; Kameyama, K.; Takeuchi, T. Value of labial salivary gland histopathology for diagnosis of Sjögren's syndrome in patients with anti-centromere antibody positivity. *Int J Rheum Dis.* **2020**, *23*, 1024–1029.
